# Understanding the role of neutrophils in chronic inflammatory airway disease

**DOI:** 10.12688/f1000research.18411.1

**Published:** 2019-04-26

**Authors:** Alice E Jasper, William J McIver, Elizabeth Sapey, Georgia M Walton

**Affiliations:** 1 Birmingham Acute Care Research, Institute of Inflammation and Ageing, University of Birmingham, UK, Birmingham, B15 2TT, UK

**Keywords:** Neutrophil, COPD, Asthma, Cystic Fibrosis, Bronchiectasis, Alpha-1 Anti-Trypsin, Inflammation

## Abstract

Airway neutrophilia is a common feature of many chronic inflammatory lung diseases and is associated with disease progression, often regardless of the initiating cause. Neutrophils and their products are thought to be key mediators of the inflammatory changes in the airways of patients with chronic obstructive pulmonary disease (COPD) and have been shown to cause many of the pathological features associated with disease, including emphysema and mucus hypersecretion. Patients with COPD also have high rates of bacterial colonisation and recurrent infective exacerbations, suggesting that neutrophil host defence mechanisms are impaired, a concept supported by studies showing alterations to neutrophil migration, degranulation and reactive oxygen species production in cells isolated from patients with COPD. Although the role of neutrophils is best described in COPD, many of the pathological features of this disease are not unique to COPD and also feature in other chronic inflammatory airway diseases, including asthma, cystic fibrosis, alpha-1 anti-trypsin deficiency, and bronchiectasis. There is increasing evidence for immune cell dysfunction contributing to inflammation in many of these diseases, focusing interest on the neutrophil as a key driver of pulmonary inflammation and a potential therapeutic target than spans diseases. This review discusses the evidence for neutrophilic involvement in COPD and also considers their roles in alpha-1 anti-trypsin deficiency, bronchiectasis, asthma, and cystic fibrosis. We provide an in-depth assessment of the role of the neutrophil in each of these conditions, exploring recent advances in understanding, and finally discussing the possibility of common mechanisms across diseases.

## Introduction

**Figure 1.**
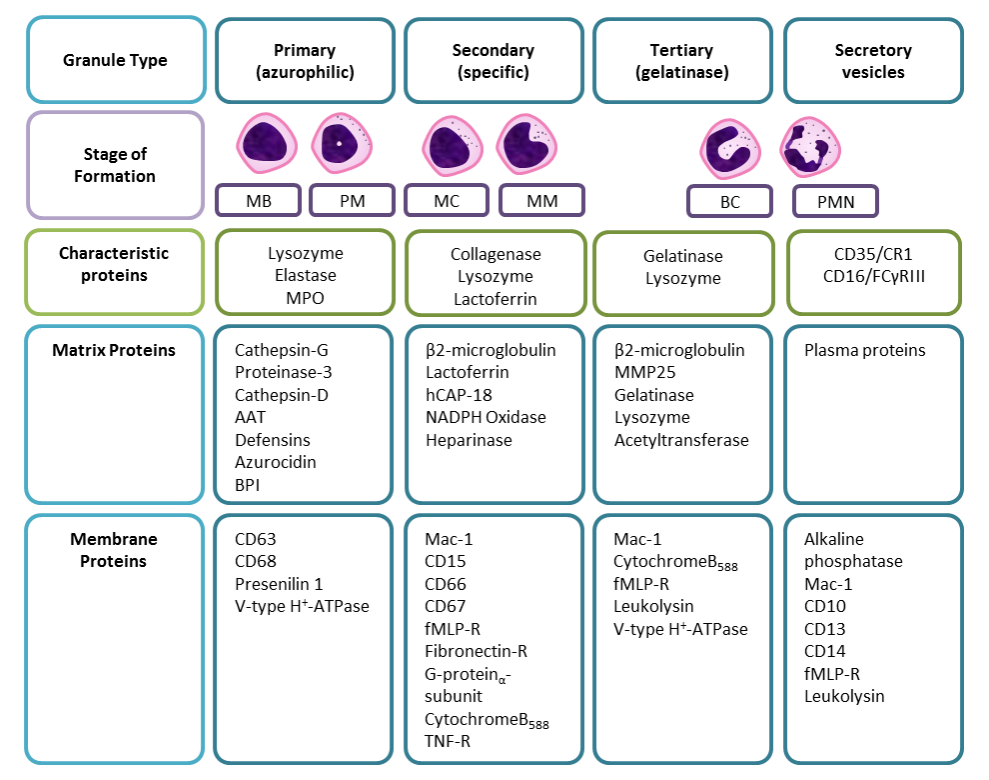
The contents of neutrophil granule subtypes split into characteristic, matrix (cytosolic), and membrane proteins. AAT, alpha-1 anti-trypsin; BC, band cell; BPI, bacterial permeability-increasing protein; CR1, complement receptor-1; fMLP, *N*-formylmethionine-leucyl-phenylalanine; hCAP-18, human cathelicidin protein-18; Mac-1, macrophage-1 antigen (CD11b/CD18); MB, myeloblast; MC, myelocyte; MM, metamyelocyte; MMP, matrix metalloproteinase; MPO, myeloperoxidase; NADPH, nicotinamide adenine dinucleotide phosphate; PM, promyelocyte; PMN, polymorphonuclear neutrophil; R, receptor; TNF, tumour necrosis factor. Data were combined from 10– 12.

Neutrophils are the dominant circulating leucocyte, comprising around 70% of white blood cells in health and representing a key component of the innate immune system. Neutrophils are short-lived cells (with a half-life of about 8 hours), having a basal production of 1 to 2 × 10 ^11^ neutrophils per day in health, although this can increase to 10 ^12^ during infection and their half-life can also increase in the presence of inflammation and hypoxia ^1^. Neutrophils are characterised by their multi-lobed nucleus and granular cytoplasm, the latter caused by azurophillic (primary), specific (secondary) and gelatinase (tertiary) granules, as well as secretory vesicles (contents described in Figure 1). These granules and vesicles contain a complex armamentarium of products that permit cell communication, neutrophil migration, microbial killing, tissue remodelling, degradation and repair.

In response to infection, neutrophils leave the circulation and migrate to the affected sites, where they use a variety of mechanisms to contain and kill invading pathogens, preventing further dissemination. The phagocytosis of bacteria leads to intracellular pathogen killing within a contained structure (the phagolysosome) to protect the cell and surrounding tissue. The phagolysosome is formed when neutrophil granules (which contain pre-formed products such as proteinases and bactericidal proteins and newly formed reactive oxygen species [ROS]) fuse with the lysosome containing the ingested bacterium. ROS production is a convoluted process, necessary to protect the host from the free radical-associated harm. NADPH oxidase is constructed from a series of subunits and then acts as a channel for electrons from the cytosol to enter the phagolysosome, stimulating reduction of oxygen (O _2_) to the superoxide anion O _2_^−^^2^. Superoxide then can dismutate to form the highly oxidative hydrogen peroxide (H _2_O _2_), which can react further, forming the strongly bactericidal hypohalous acids (for example, hypochlorous acid) ^3– 8^. These products can also be released into the extracellular matrix by degranulation, but neutrophils require different levels of activation to release granules; secretory vesicles are released during minimal stimulation to facilitate migration and adhesion, and azurophil granules (the most cytotoxic) require the most stimulation.

Release of azurophil granules leads to areas of obligate tissue damage, as the proteinases contained therein readily digest components of the extracellular matrix until their inhibition by anti-proteinases can occur ^9^. Finally, in overwhelming infection or inflammation, neutrophils have been described as releasing their decondensed DNA in web-like structures outside of the cell. The extruded DNA is coated in cytotoxic products, including proteinases, termed neutrophil extracellular traps (NETs) as they can ensnare and “trap” bacteria ^13^. To protect the host from this immense arsenal, neutrophils are held in three states that they can fluctuate between: quiescent, primed and activated. The primed state provides both a mechanism to allow a rapid and pro-inflammatory response to infection but also a brake to prevent unwarranted degranulation ^14^.

There is increasing interest in neutrophil phenotypes or subtypes, and cells appear to have a broader repertoire of responses than the highly aggressive, cytotoxic response described above. The concept of cell phenotypes is well established with lymphocytes or macrophages but remains more controversial with neutrophils. However, despite previous theories, neutrophils are transcriptionally active ^15^, can release a wide range of context-specific products ^16^ and have an adaptable lifespan depending on activation status and environment ^17^. They also express more than 30 different receptors—including G protein–coupled receptors, Fc receptors, adhesion receptors, cytokine receptors and pattern recognition receptors—that can sense pro-inflammatory mediators and modulate neutrophil migration, function and behaviour ^18^, suggesting plasticity in their responses. There have been descriptions of pro-angiogenic neutrophils ^19^, characterised by increased matrix metalloproteinase 9 (MMP-9) release ^20, 21^ and anti-inflammatory neutrophils capable of suppressing other immune cells ^22^. Research into the exact function of these phenotypes (or indeed whether they do represent different cell types or are an adaption of the cell to environmental stimuli) remains unclear, but it might be that different subsets of cells have different functions; indeed, when neutrophils are viewed, it is clear that cells within a field behave heterogeneously ^23^.

What is evident is the immense potential that neutrophils have for host damage which requires constant check. Recently, it has been suggested that the lung may be involved in this process. Neutrophils are larger in diameter than some of the tortuous pulmonary vasculature that they must traverse. Initially, it was considered that the lungs hold a sequestered pool of neutrophils, slow in transit through the capillary bed and ready to respond to pulmonary infection ^24– 26^. However, recent research has shown that this is not the case in health, and there is no evidence of a retained neutrophil population in the lungs unless there is systemic priming of neutrophils and even this results in only a transitory retention of neutrophils ^27^. It was then demonstrated that the process of manoeuvring through tight spaces promoted neutrophil de-priming ^28^, leading to the hypothesis that the sinuous pulmonary vasculature might be a site where the primed neutrophil population (thought to be up to 40% of the whole population in some studies) can be “stood down” into a quiescent state ^29^.

Perhaps then it is no surprise that there is evidence of neutrophil dysregulation during lung disease, that airway neutrophilia is a feature of multiple lung pathologies and that patients with airway disease often display heightened and more damaging neutrophilic inflammation. This may represent a physiological response to an infective or inflammatory trigger (such as neutrophil recruitment to the lung in response to a respiratory infection or cigarette smoke) or a physiological response to a pathological environment (the inflamed and damaged lung causing increased neutrophil recruitment and being less able to “de-prime” cells). However, there is amassing evidence that the neutrophil itself may inflict further harm to the host by intrinsic changes to key cellular functions. Understanding whether the neutrophil is a reactive responder or a creative actor in lung disease (or indeed both) is vital when considering the development of new therapeutics: would one target the environment or the neutrophil? The evidence for these processes in airway disease has been most thoroughly described in chronic obstructive pulmonary disease (COPD). This review will explore what is currently known about neutrophils in the pathogenesis of airway disease, focusing mainly on COPD. However, neutrophil function in alpha-1 anti-trypsin deficiency (AATD), bronchiectasis, cystic fibrosis (CF) and asthma will also be considered in order to assess the likelihood of common mechanisms and therefore potential therapies which could span diseases.

## Chronic obstructive pulmonary disease

COPD is a leading cause of morbidity and mortality worldwide and constitutes a significant healthcare burden ^30– 32^. In the UK, chronic cigarette smoking remains the largest cause of COPD, but after 25 years of smoking, only about 30 to 40% of adults will have developed COPD ^33^, and COPD is diagnosed in never smokers (who may have other environmental exposures which lead to disease) ^34^, suggesting that smoking is neither necessary nor sufficient to cause COPD.

Airway inflammation is central to the pathophysiology of COPD and contributes to tissue damage and destruction and a wealth of data support a role for the neutrophil at the heart of this inflammatory process. All patients with COPD have airway neutrophilia, regardless of clinical phenotype (chronic bronchitis, emphysema, and even eosinophilic COPD), disease severity, and rate of decline or age of onset. COPD is very heterogeneous and although patients may share a cause (such as cigarette smoking), the disease presentation is variable, suggesting that COPD is more an umbrella term than a narrow clinical entity ^35^ ( Table 1). Neutrophil numbers (and their products) relate to airway obstruction, decline in forced expiratory volume in 1 second (FEV _1_), reduction in gas transfer, and development of emphysema ^36– 40^. Although patients with COPD demonstrate airway neutrophilia, they also experience airway colonisation and recurrent bacterial infections ^41– 44^. This raises the possibility that the function of neutrophils is impaired, leading to reduced anti-microbial function and at the same time contributing to lung damage and a number of observations support this concept.

**Table 1.** Recognised clinical phenotypes of chronic obstructive pulmonary disease, asthma, and bronchiectasis.

Phenotype	Basic features
**Chronic obstructive pulmonary disease (COPD)**	
Bronchitic phenotype	The presence of productive cough (at least 3 months per year in at least 2 consecutive years)
Emphysema phenotype	Presence of emphysema confirmed on imaging (including computed tomography densitometry)
Eosinophil COPD	Presence of eosinophilia, normally defined as at least 2% eosinophils in either blood or sputum
Asthma COPD overlap	Persistent airflow limitation with several features usually associated with asthma and several features usually associated with COPD
Overlap COPD and bronchiectasis	Airflow obstruction consistent with COPD alongside irreversibly dilated airways, mucus gland hyperplasia and impaired mucus clearance associated with bronchiectasis
Frequent exacerbation phenotype	Two or more “exacerbation” events per year; an exacerbation is defined as an acute worsening of respiratory symptoms that result in additional therapy.
**Asthma**	
Atopic asthma	Atopic and eosinophilic with increased fractional exhaled nitric oxide (FeNO)
Non-eosinophilic asthma associated with obesity	Decreased lung function associated with obesity
Non-eosinophilic asthma (neutrophilic asthma)	Lack of eosinophilic inflammation. No raised sputum eosinophil count or FeNO. Neutrophilic inflammation common.
**Aetiology**	**Examples of causes**
**Bronchiectasis**	
Post-infectious damage	Tuberculosis, whooping cough, and so on
Muco-ciliary clearance defects	Primary ciliary dyskinesia, cystic fibrosis, and Young’s syndrome
Immunodeficiency	Primary (for example, hypogammaglobinaemia) Secondary (for example, malignancy such as leukaemias or immune modulation with drugs, after transplant)
Autoimmune conditions	Rheumatoid arteritis, systemic lupus erythematosus, and inflammatory bowel disease
Congenital	Tracheobronchomegaly, cartilage deficiency, and Marfan syndrome
Toxic exposures, obstruction or aspiration	Toxic gas (chlorine, ammonia), foreign body, and smoke exhalation

The first and second sections provide a table of recognised COPD and asthma phenotypes. Though not exhaustive, these represent phenotypes most often discussed in recent publications (for example, 47, 48) and it is also possible for patients to have more than one phenotype; thus, there can be considerable clinical overlap. In the third section, examples of aetiologies that can lead to bronchiectasis have been given. Again, this list is not exhaustive but for all diseases (COPD, asthma and bronchiectasis) is intended to provide an overview of how disparate clinical phenotypes associated with one umbrella term can be.

### Neutrophil migration and chronic obstructive pulmonary disease

Older studies of neutrophil migration in COPD yielded conflicting results as to whether there was any compromise in migratory function ^45, 46^; however, more recent studies have allowed the assessment of specific neutrophil migratory dynamics and have shown that neutrophils from patients with COPD migrate with increased speed but reduced directional accuracy towards a variety of chemoattractants compared with age-matched healthy control subjects ^40^. This does not result from reduced chemoattractant receptor expression or impaired receptor localisation ^49^ but could be improved by using a broad-spectrum PI3K inhibitor (LY294002‎), suggesting that the defective migration results from aberrant intracellular signalling processes ^40^.

As a neutrophil migrates, serine proteinases, including neutrophil elastase (NE), cathepsin G and proteinase 3 (PR3), are released from azurophil granules into the extracellular space, and some active enzyme is retained on the plasma membrane ^9, 50– 52^. Substrates for these proteinases include elastin ^53^, collagen ^54^ and fibronectin ^55^, which are major components of the extracellular matrix, and their degradation is linked to all clinical facets of COPD.

Initially, NE was thought to be the most important proteinase in COPD. NE can be inhibited by a number of endogenous inhibitors, including alpha-1 anti-trypsin (AAT), secretory leucocyte proteinase inhibitor (SLPI) and α2-macroglobulin (α2M). However, at the time of NE release, its concentration (5.33 mM) is 15 to 1500 times greater than that of its inhibitors ^56^ (with plasma concentrations of AAT of 32.8 μM ^9^, SLPI of 11 μm ^57^ and α2M of 3.5 μM ^58^). NE is only partly inhibited until it diffuses away from the cell and an optimal NE-inhibitor ratio is reached. As this inhibition is not immediate, an obligate area of local, proteinase-mediated tissue damage occurs, a phenomenon known as “quantum proteolysis”. Furthermore, a proportion of NE remains bound to the neutrophil cell surface, making it less accessible to inhibitors and more resistant to inhibition, further increasing the local potential damage. There is emerging evidence that PR3 may be even more implicated in the pathology of COPD, especially the emphysema process ^59^. PR3 is stored in higher concentrations than NE ^60^ and has a lower association rate constant with AAT, suggesting that it is likely to have more prolonged activity than NE before inactivation, as demonstrated by mathematical modelling ^61^ and in studies of airway secretions in both AAT-deficient (AATD) and non-deficient COPD ^62^.

Reduced migratory accuracy of neutrophils in COPD may have implications for disease pathology because of the increased area of obligate tissue damage caused by proteinase release during poorly directed migration. In keeping with this, previous work has shown increased fibronectin degradation by migrating COPD neutrophils ^45^; *in vitro*, there are increased levels of the NE footprint Aα-Val ^360^ ^63^ and newly described PR3 footprint Aα-VAL ^541^ ^64^ in plasma from patients with COPD.

Of note, recent studies suggest that migration of monocytes from COPD patients to COPD sputum is also impaired ^65^, although similar studies using single chemokines as the chemoattractant do not replicate this finding ^66^. As neutrophils and monocytes are derived from the same bone marrow precursor cells, this may reflect a common genetic/epigenetic or inflammatory cause. It remains unclear when neutrophil migratory dysfunction develops in COPD: during maturation in the bone marrow or following release into the circulation. If monocyte migration were found to be similarly impaired in COPD, this might suggest that the defect lies in bone marrow precursor cells.

COPD is more commonly seen in older patients and neutrophil migratory accuracy also declines with “healthy” ageing, a phenomenon also associated with constitutive PI3K activity ^67^, and selective class I PI3K-δ or PI3K-γ inhibitors improve neutrophil migratory accuracy. It is possible that the poor migratory accuracy seen with age is exaggerated in patients with COPD, and inhibition of specific PI3K isoforms may offer a novel strategy to improve bacterial clearance and reduce tissue damage in COPD.

### Proteinases and chronic obstructive pulmonary disease

The proteinase/anti-proteinase theory of COPD suggests that damage to the lung tissue occurs when the levels of anti-proteinases in the lung are insufficient to effectively neutralise the proteinases present ^62, 68, 69^. Recently, the proteinase/anti-proteinase theory of COPD was revisited in a study which elegantly demonstrates a role for exosomes (cell-derived vesicles that are present in many eukaryotic fluids, including blood, urine and cerebrospinal fluid) in promoting NE activity, effectively tipping the local protease/anti-protease balance within the lung to favour tissue damage ^70^. The authors describe a population of exosomes released from neutrophils which bind NE when it is released during degranulation, before its diffusion into the tissues. Unlike free enzyme, NE bound to these exosomes was found to be resistant to inhibition by AAT and to be able to bind to the extracellular matrix (via mac-1) whilst maintaining NE activity against the extracellular matrix proteins. These neutrophil-derived exosomes were found in clinical specimens from subjects with COPD but not healthy controls and importantly were capable of transferring a COPD-like phenotype from humans to mice ^70^. Certainly, there is evidence of increased degranulation in COPD, and CD63 (a marker of primary granules) is found to be increased on the surface of unstimulated neutrophils from patients with COPD ^71^.

Serine proteinases are potent stimulators of mucus secretion from submucosal and goblet cells of the airways ^72^, which, alongside the effects of cigarette smoke on mucosal cilia, reduces mucociliary clearance ^73, 74^. Mucus is able to build up in the airways, contributing to further obstruction, increasing the risk for bacterial colonisation and further inflammation ^72, 75^.

### Neutrophil extracellular traps and chronic obstructive pulmonary disease

Recently, there has been significant interest in the role of NETs in COPD, but the current evidence is conflicting. Increased quantities of NET components have been described in the sputum of both stable and exacerbating COPD patients, alongside an increased proportion of “NET producing” neutrophils ^76, 77^. Furthermore, the abundance of NETs within sputum has been shown to correlate with severity of airflow limitation assessed by FEV _1_^76, 78^ and overall severity of COPD using a composite scale including symptoms and exacerbation frequency alongside FEV _1_^78^. Interestingly, the most recent study of NETs in COPD shows a correlation between NET complexes in sputum and microbial diversity, in particular a dominance of haemophilus species, whereby more than 40% haemophilus species within the lung microbiome were found to be associated with significantly greater DNA-elastase complexes ^78^. Despite this, neutrophils isolated from the blood of patients with exacerbations of COPD have been shown to have a reduced ability to undergo NETosis compared with both stable patients and healthy controls, despite the increased presence of cell-free DNA in plasma ^79^. This seems counterintuitive but it is possible that the clearance of NETs by DNases ^80^ is impaired in COPD or that only a proportion of cells are able to produce NETs (a phenotype of cell) but this remains unknown.

### Phagocytosis and chronic obstructive pulmonary disease

Reduced phagocytic function of macrophages in COPD is well described, encompassing impaired phagocytosis of disease-relevant bacteria (non-typeable *Haemophilus influenzae* and *Streptococcus pneumoniae*), fungi ^81^ and apoptotic neutrophils via efferocytosis ^82– 84^. However, little research to date has focused on the phagocytic ability of neutrophils, and data so far provide conflicting results; some demonstrate no differences in phagocytosis ^85– 87^ and others suggest a reduction ^88^. However, few studies have used phagocytic targets relevant to disease pathology, and studies in macrophages suggest that the use of non-physiological targets may provide misleading results. For example, phagocytosis of synthetic beads by macrophages was found to be unaltered in COPD, but bacterial studies showed a reduction in phagocytosis of the disease-relevant bacteria *H. influenzae* and *S. pneumoniae* ^82^, suggesting that the results of studies using non-physiological targets may be misleading ^85, 88^. This gap in knowledge clearly needs detailed exploration.

Further to this, NE has been shown to be able to cleave complement components on bacteria as well as complement receptors on neutrophils ^89, 90^. If NE activity is heightened, as suggested in COPD ^91^ and AATD ^92^, the resulting opsonin-receptor mismatch may impair effective phagocytosis.

### Neutrophil phenotypes in chronic obstructive pulmonary disease and retention in the lung

Despite emerging interest in the concept of neutrophil phenotypes, there are few studies of this in COPD. One recent article assessed protein expression on the surface of neutrophils from 41 patients with COPD and seven healthy, age-matched controls, describing clear clustering which could differentiate patients with COPD from the control subjects ^93^. Furthermore, the neutrophil proteome was different between two COPD groups; but these patient groups were not clinically different, the expressions of several activation markers were not significantly different, but there were some functional changes between groups in relation to ROS release ^93^.

In relation to the retention of neutrophils in COPD lungs, a very recent publication ^94^ has built upon the previously described studies of neutrophil transit times through the lung vasculature, clearly demonstrating increased neutrophil accumulation in COPD lungs compared with healthy individuals, with little overlap.

In summary, recent studies in COPD have built upon a strong foundation implicating the neutrophil as a key driver of COPD pathology. This includes altered cell functions which favour host tissue damage with an increased burden of proteinase activity, a clear signal of neutrophil retention in the lungs which is not seen in health, and a tantalising hint of differing cell phenotypes. However, because COPD studies invariably compare health with disease, it is unclear whether these changes are unique to COPD (and thus may represent a COPD-specific therapeutic target) or whether these changes are also seen in other diseases of the airways.

## Alpha-1 anti-trypsin deficiency

AAT is a 52-kDa glycoprotein produced by hepatocytes but also macrophages and neutrophils and (as stated) functions as a serine protein inhibitor, providing essential protection of the lung tissue against the proteolytic actions of enzymes such as NE and PR3. In health, there is a constant diffusion of AAT into the lung, which is increased in the presence of inflammation (such as during respiratory infections). AATD is a genetic disorder in which the gene encoding AAT is mutated. There are many subtypes of AATD but in the most common severe form of deficiency (named PiZZ) this leads to mis-folding of the protein product, retention of AAT in AAT-producing cells and the formation of protein polymers in these cells, which causes damage and low circulating levels of AAT. AATD is the only robustly established genetic risk factor for the development of COPD and emphysema, and these disease processes can occur in patients with AATD, even in the absence of cigarette smoking ^62, 91, 95, 96^.

Neutrophils play a central role in the pathophysiology of emphysema associated with AATD ^97^, and pulmonary disease is thought to develop, in part, from an imbalance of proteinases and AAT, although AAT has many non-proteolytic functions which protect against infection and inflammation, including immunomodulation and anti-microbial activity. It is well known that AAT deficiency is associated with a reduced ability to neutralise NE and PR3 adequately, leading to more tissue damage. In response to NE activity, epithelial cells and macrophages also release pro-inflammatory mediators such as CXCL8 ^98^ and leukotriene B _4_ (LTB _4_) ^99^, respectively. This chemoattractant production is perpetuated, further increasing neutrophil influx and increased NE activity within the lung, forming a vicious cycle of damage. In keeping with this, the inflammation present in AATD (both systemic and local) is amplified when compared with non-AATD COPD with a similar burden of disease ^100^ and this may influence immune function by cell priming or activation.

Lung neutrophilia has been much easier to demonstrate in AATD compared with non-AATD COPD ^101^ but these cells do not appear to be just “reactive responders”, and a number of studies have described abnormal neutrophil behaviour in AATD. A recent study described increased apoptosis of AATD neutrophils ^102^. The authors proposed that this might reflect endoplasmic reticulum stress owing to the accumulation of mis-folded AAT within the neutrophil ^102^. However, augmentation therapy, in which deficient AAT is “replaced” with purified plasma AAT from healthy individuals, was able to normalise cell apoptosis without altering endoplasmic reticulum stress markers, and apoptosis was a direct result of low circulating AAT. Internalised AAT is known to co-localise with and inhibit staurosporine-induced caspase-3 activation ^103^, a potent signal for apoptosis recently described in neutrophils ^104^.

The same authors propose defective bacterial killing by AATD neutrophils but this appeared to result from accelerated neutrophil apoptosis rather than an intrinsic defect in neutrophil phagocytosis *per se* ^102^. AAT augmentation both *in vitro* and *in vivo* could restore the bacterial killing capacity of ZZ-AATD neutrophils to that of non-deficient neutrophils but again this might reflect reduced apoptosis ^102^. AAT is known to improve phagocytosis by both human alveolar macrophages (AMs) from patients with non-AATD COPD and AMs isolated from mice exposed to cigarette smoke ^105^. This improvement included both efferocytosis (clearance of dead neutrophils) and phagocytosis and was associated with the upregulation of efferocytosis and scavenger receptors on the AM plasma membrane ^105^. These receptors were also shown to be upregulated in patients with AATD following double-dose augmentation treatment with purified AAT compared with a single dose, suggesting that a similar mechanism to enhance efferocytosis may exist *in vivo*.

There are limited data regarding neutrophil migratory function in patients with AATD. One study has suggested that sputum from patients with AATD has greater chemotactic activity which likely relates to increased levels of neutrophil chemoattractants CXCL8 and LTB _4_ in sputum rather than the ability of neutrophils to migrate *per se* ^106^. A further study demonstrated no difference in the ability of neutrophils from patients with AATD to migrate to a standard chemoattractant (CXCL8) compared with healthy controls despite finding reduced migratory accuracy of neutrophils from patients with COPD ^40^. There is an increased burden of ROS in AATD, and AAT modulates neutrophil O _2_^−^ production elicited by *N*-formylmethionine-leucyl-phenylalanine (fMLP) and CXCL8 in a dose-dependent manner ^107^. However, the burden of ROS in AATD may be multi-faceted, and AAT is known to bind to a number of products with oxidative potential, including hemin ^108^. There are few studies of NET formation in AATD. One study using the non-physiological stimulant phorbol myristate acetate (PMA) reported that AAT did not reduce NET formation from neutrophils isolated from patients with PiZZ AATD but this study has yet to be replicated using disease-relevant stimuli ^109^.

To date, there are no studies of neutrophil phenotype in PiZZ AATD to determine whether distinct patterns are seen, but when the data are considered together, it appears that AATD is not merely an exaggerated form of non-AATD COPD and there appear to be differences in cellular function between the two groups. This is highlighted in clinical and imaging studies. The predominant phenotype of emphysema observed in non-AATD COPD is typically apical centrilobular but this differs in AATD, where emphysema is predominantly basal and panlobular ^110, 111^, reflecting differences in pathophysiology between the two conditions. 18-fluorodeoxyglucose ( ^18^FDG) positron emission tomography–computed tomography (PET-CT) studies generate both quantitative and spacial data regarding pulmonary glucose uptake, which has been shown to relate to neutrophil activity in animal models ^112, 113^. When these studies were performed in patients with COPD, ^18^FDG uptake was shown to be greater in emphysematous regions of the lung and correlated with physiological measures of disease severity ^114^. Despite this, the increased pulmonary ^18^FDG demonstrated in non-AATD COPD was not demonstrated in a small cohort of patients with AATD, in whom ^18^FDG uptake was comparable to that of healthy controls ^114^. This suggests important differences in the pathogenesis of emphysema in these two conditions, in particular with respect to the role of the neutrophil.

Also, lung disease is heterogeneous in AATD, even in patients with PiZZ AATD who have never smoked. A proportion of never-smoking patients with similar deficiency levels do not develop lung disease although some do, and in those who do, rates of decline are variable and currently cannot be predicted at baseline screening ^115^. Furthermore, patients with AATD experience clinical phenotypes similar to those of patients with non-AATD COPD ^115^. Also, although AAT augmentation reduces the progression of lung disease in some patients, it has little impact on others, highlighting the fact that replenishing the deficient anti-proteinase is not enough to treat disease and more studies are needed to assess the utility of targeting the neutrophil in conjunction with augmentation strategies.

## Bronchiectasis

Rather than being a pathological entity (such as AATD), bronchiectasis is a chronic lung condition caused by a number of pathological insults ( Table 1) characterised visually by irreversibly dilated airways, mucus gland hyperplasia, and impaired mucus clearance resulting in recurrent severe infections and further airway damage as described by Cole’s vicious cycle hypothesis ^116– 118^. Bacterial colonisation with potentially pathogenic micro-organisms is extremely common, and neutrophils are thought to play a fundamental role in bronchiectasis pathogenesis, partially in response to this infection. Impaired mucus clearance and recurrent infections cause rich sputum levels of potent neutrophil chemoattractants, including interleukin 1 beta (IL-1β), tumour necrosis factor alpha (TNFα), CXCL8, and LTB _4_^119^. Consequently, neutrophils dominate cell populations in both the sputum and bronchoalveolar lavage fluid of patients with bronchiectasis, and neutrophil counts positively correlate with bronchiectasis disease severity ^117, 120^. This heightened inflammation impacts on neutrophil function. Systemic neutrophils from individuals with bronchiectasis have a higher level of baseline activation compared with healthy individuals, as indicated by increased CD62L and CD11b ^120^. Furthermore, blood neutrophil viability is significantly prolonged because of delayed apoptosis (a feature of inflammation) and these neutrophils release more myeloperoxidase (MPO) when unstimulated in more severe forms of the disease (suggesting constitutive priming and activation) ^120^. Systemic neutrophils seem to retain their phagocytic and anti-microbial ability compared with airway counterparts ^120^. Airway neutrophils in bronchiectasis exhibit impaired phagocytosis of pathogens, including *Pseudomonas aeruginosa* (PAO1), contributing to recurrent infections ^120^. However, this appears to improve with antibiotic therapy ^120^. In a study of 103 adults with bronchiectasis, the most frequent immune cell abnormality was reduced neutrophil oxidative burst ^121^ but there was significant heterogeneity. A comprehensive screen of immune function confirmed that 13 subjects had low levels of IgG3, six had low levels of B-cell lymphocytes and seven had low T-helper cell lymphocytes when compared with controls. All subjects had a normal neutrophil phagocytic function, but 33 of the subjects had an oxidative burst that was below that seen in health ^121^. In addition, airway neutrophils in bronchiectasis exhibit higher necrosis and impaired cell death as well as reduced clearance by macrophages, delaying inflammation resolution and causing persistent inflammation and further airway damage ^116, 120^. Furthermore, increased neutrophil degranulation causes further airway damage and correlates with worse clinical outcome ^119, 122^.

Although these studies highlight themes of neutrophil function and dysfunction across bronchiectasis, the diverse causes of disease may display different patterns. For example, primary ciliary dyskinesia (PCD) is a rare genetic disease caused by abnormal structure or function of motile cilia (or both) which leads to bronchiectasis ^123^. Recently, neutrophils from patients with PCD have been shown to display reduced migration toward CXCR2 ligands (CXCL5 and CXCL8) but not to LTB _4_ and complement component 5a. The reduced response to CXCL8 was observed in all subgroups of patients with PCD and correlated with lung function, and CXCR2 expression was downregulated on the cell surface in about 65% of the patients with PCD ^124^. However, in non-PCD bronchiectasis, neutrophil migration appears preserved ^125^, and a trial of a CXCR2 antagonist given orally for 28 days resulted in about a 70% decrease in the percentage of sputum neutrophils, suggesting that CXCR2 ligands were strong drivers of neutrophil accumulation in the lung ^126^.

The combination of infection and inflammation suggests that both anti-microbial and anti-inflammatory agents might help with disease management. Currently, the two main treatment options for bronchiectasis involve physiotherapy for clearance of mucus and antibiotics for treatment of infections ^127^, a strategy that has not changed since bronchiectasis was first characterised in the 1950s. Despite advances in understanding the pathophysiology of bronchiectasis, the rates of exacerbation and mortality amongst patients with bronchiectasis have shown little improvement ^128^. Therefore, further research is needed to understand how to prevent disease progression and to develop therapeutic targets accordingly but this may require better stratification of the cause of bronchiectasis and a diverse treatment strategy.

## Cystic fibrosis

CF is an autosomal recessive disease whereby a loss-of-function mutation in the CF transmembrane conductance regulator ( *CTFR*) gene affects mucociliary clearance ^117^. CF is the most common inherited disorder in the Caucasian population, affecting 1 in 2000 live births ^129^, and a common cause of bronchiectasis, which is often more severe and progressive than non-CF bronchiectasis. With CF bronchiectasis, as with non-CF bronchiectasis, neutrophil dysfunction has been described. In particular, airway neutrophils in patients with CF exhibit a functional exhaustion and a pro-survival phenotype ^129, 130^, potentially reflecting the high levels of inflammation and structural damage present in the lung.

Recruitment and migration of neutrophils into the lung appear to be normal in patients with CF, but the plethora of inflammatory mediators in the CF airways makes the sputum rich in neutrophils ^117^. Extensive research into these airway neutrophils has uncovered some functional defects. First, CF airway neutrophils exhibit impaired degranulation which is linked to the loss of CFTR function as ivacaftor treatment reverses this ^129, 131^. Dysregulated degranulation of NE and MPO contributes to tissue damage which can exacerbate CF ^117^. Furthermore, the altered microenvironment of the CF lung is thought to be a contributing factor to lower neutrophil phagocytosis levels which are coupled with a lower respiratory burst generation shown *in vitro* following stimulation with PMA ^117, 130^. This is thought to contribute to impaired bacterial killing and recurrent infections. In addition to having functional defects, CF airway neutrophils appear to have a pro-survival phenotype. CF neutrophils have apoptosis defects which delay and impair cell death, resulting in neutrophil persistence, NET production and increased necrosis ^129, 130, 132, 133^. Auto-antibodies to NET components have also been described in patients with CF and the presence of these auto-antibodies has been associated with diminished lung function ^134^, although direct evidence linking these two observations is lacking.

New CFTR modulators have revolutionised the treatment of CF for patients with specific genetic mutations and these therapies also appear to impact on neutrophil function. Ivacaftor treatment restored neutrophil apoptosis rates in patients commencing this treatment compared with their baseline functions ^129, 131^. The exact mechanism of action has not yet been elucidated, but similar immune modification has been seen in macrophages from CF patients taking CFTR modulators ^135^, providing further evidence of effect.

## Asthma

Asthma is a chronic inflammatory lung disease that affects 340 million people worldwide and accounts for 180,000 deaths worldwide every year ^136, 137^. Again, there are many phenotypes of asthma ( Table 1), and for many years there has been interest in the concept of “neutrophilic asthma” (where neutrophils represent 40 to 76% of total sputum cells) and this classically correlates with steroid resistance, acute exacerbations, occupational asthma and more treatment-resistant forms of the disease, suggesting that the neutrophil plays a role in asthma pathophysiology ^136, 138– 140^. In patients with asthma, as in those with other diseases discussed, both peripheral and airway neutrophils exhibit functional defects compared with healthy individuals. *In vitro* chemotactic velocity to CXCL8 and fMLP has been shown to be impaired ^137^. This finding has led to the suggestion that neutrophil migration could be used to differentiate asthma from non-asthma patients ^141^, but given that neutrophil migration is dysfunctional in a number of conditions, the utility of such a device is questionable. In asthma, as in other airway diseases, there is some evidence of increased NET formation ^142^, ROS generation ^143^ and reduced neutrophil phagocytosis ^144^, although results are variable. In patients with neutrophilic asthma, as in those with COPD, systemic inflammation (C-reactive protein and IL-6) is increased compared with both patients with non-neutrophilic asthma and healthy controls ^145^. However, whether defects in neutrophil function are intrinsic or are a consequence of—and perhaps contribute to—heightened systemic and airway inflammation remains unclear.

Of note, although neutrophils are associated with tissue damage in asthma, they have also been shown to have a role in controlling inflammation, restoring tissue homeostasis and promoting tissue repair ^136^, highlighting the delicate balance between protective and destructive functions of neutrophils in airway disease, a common feature across all of the diseases we have considered.

## Common mechanisms across diseases

The data presented highlight many similarities in neutrophilic inflammation across airway diseases. First, an airway neutrophilia is common. Second, there are often markers of neutrophil degranulation and in particular ROS and proteinase activity which are associated with disease presentation and progression. Third, aspects of neutrophil function appear altered. Although this seems to most commonly affect migration and ROS production, studies also highlight aberrant NETosis and phagocytosis, although defects are variable. The commonality of neutrophil inflammation across different diseases might suggest common underlying mechanisms of effect, and studies have suggested potential themes as to how this might occur.

**Figure 2.**
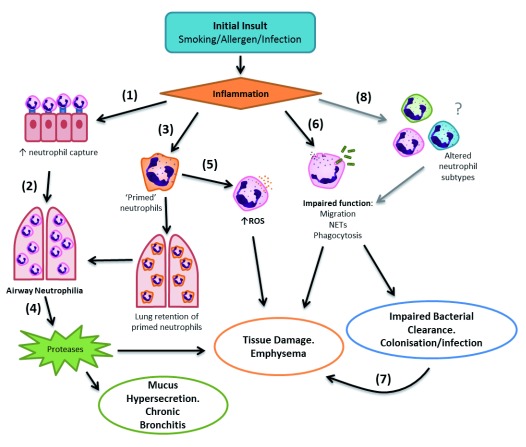
Inflammatory mechanisms in disease pathogenesis. Inflammation from the initial insult **(1)** increases the expression of capture molecules on the bronchial epithelium and adhesion molecules on neutrophils, **(2)** enhancing neutrophil migration into the inflamed lung, resulting in airway neutrophilia. **(3)** Potentially altered neutrophil priming processes from excessive neutrophil priming, or a possible failure of the lung to “de-prime” neutrophils, further increases airway neutrophilia. **(4)** Release of proteases from airway neutrophils during migration, release of neutrophil extracellular traps (NETs), or frustrated phagocytosis contributes to degradation of elastin and development of emphysema. Neutrophil elastase can also cause mucus hypersecretion, contributing to development of chronic bronchitis. **(5)** Increased reactive oxygen species (ROS) released from primed neutrophils further contributes to tissue damage within the lung. **(6)** Impaired neutrophil function increases tissue-damaging potential via excessive protease release or impaired bacterial clearance, increasing susceptibility to bacterial colonisation or acute infection. **(7)** Bacterial colonisation further heightens pulmonary inflammation, increasing tissue damage potential. **(8)** Speculatively, inflammation, hypoxia or physical pressure may alter the neutrophil population, resulting in subtypes of neutrophils with different phenotypes and altered function which further contribute to local tissue damage and impaired bacterial clearance.

First, inflammation might impact on neutrophil functions irrespective of the initial insult (be it infection, allergen or smoking). For example, TNFα—shown to be increased in airway secretions from patients with COPD, asthma, bronchiectasis and AATD—is able to increase the expression of capture receptors and adhesion molecules on the surface of blood vascular endothelial cells, enhancing neutrophil migration into the inflamed lung ^146^. Furthermore, TNFα can impact on cellular functions as it is a potent priming agent and able to increase ROS production by neutrophils, which will further contribute to tissue damage ^147^. Second (and more speculatively), the inflammation present across diseases might impair the ability of the lungs to “de-prime” cells ^29^, leading to a circulating population of primed cells, which might confer a more aggressive cellular phenotype. A third putative theme is that of altered cellular subtypes. This has only recently been described in COPD, but other studies suggest that neutrophils change in response to signals such as inflammation, hypoxia or physical pressure, resulting in different functional phenotypes. These changes are subtle and may relate to the immediate cellular environment, as described in mice models of cancer ^147^ and pro-inflammatory culture conditions ^148^. Figure 2 provides a summary of how these mechanisms could lead to pathology across diseases. The evidence base for these themes is tentative but, if confirmed, may provide therapeutic insight to target fundamental inflammatory processes across diseases.

## Targeting neutrophils in airway disease

Although modifying neutrophilic inflammation is an attractive interventional strategy, neutrophils are a challenging target and one that comes with risks associated with neutropenia or excessive inhibition of neutrophil host defence mechanisms against infection. There are two potential therapeutic avenues to explore.

The first is to target the chemoattractants responsible for neutrophil recruitment into the lung. CXCL8 and LTB _4_ are the dominant chemokines thought to be responsible for this ^100, 106, 149^ and drugs targeting these mediators have been trialled in inflammatory airway disease. Clinical studies of a CXCR2 agonist (MK-7123) led to improvements in lung function and reduced exacerbations in active-smoking patients with COPD compared with placebo treatment ^150^, but a large proportion of patients experienced neutropenia, raising concerns about immunosuppression. As discussed earlier, in patients with bronchiectasis, the CXCR2 antagonist AZD5069 resulted in a 70% decrease in the percentage of sputum neutrophils but this was not associated with improved clinical outcomes ^126^. A small phase II trial investigated the effects of a leukotriene synthesis inhibitor, reducing LTB _4_ production, on bronchial inflammation in patients with stable COPD, showing some benefit ^151^. However, a randomised placebo-controlled trial of an LTB _4_ receptor antagonist (BIIL 284 BS) in patients with CF was terminated early because of serious adverse effects, including increased respiratory symptoms requiring intravenous antibiotics and hospitalisation, reduced pulmonary function and increased circulating neutrophil numbers ^152^, suggesting that LTB _4_ antagonism may result in acute pulmonary exacerbations and heightened inflammation ^152^, although the mechanisms for this were poorly understood. An alternative strategy may be to target associated co-morbid conditions which contribute to the inflammatory load. For example, the treatment of periodontitis, a chronic inflammatory condition associated with neutrophilic inflammation and recruitment which shares many inflammatory features of COPD ^153^, has been shown to improve changes in both lung function and exacerbation frequency in COPD ^154^.

The second therapeutic option is to directly modulate neutrophil function ^155^. A number of *in vitro* studies have investigated strategies to improve the accuracy of neutrophil migration, thereby theoretically reducing the potential for migration-associated and protease-mediated tissue damage. Broad-range inhibition of PI3K signalling has been shown to restore migration of COPD neutrophils to levels similar to those of neutrophils from age-matched healthy controls ^40^. However, broad-spectrum inhibition of PI3K therapeutically is likely to lead to significant side effects. Selective inhibition of class I PI3K-δ and PI3K-γ, which are enriched in leucocytes ^156^, may offer a more acceptable therapeutic option; indeed, selective isoform inhibition of PI3K-δ and PI3K-γ has been shown to restore reduced migratory accuracy of neutrophils from healthy older adults ^67^. Impaired migration in COPD is hypothesised to be a further exaggeration of age-related impairment in neutrophil migration, emphasising the potential of this strategy in COPD, but whether PI3K inhibition provides benefit *in vivo* or in other inflammatory airway diseases requires further investigation. Simvastatin is a safe and well-tolerated drug commonly used for its cholesterol-lowering abilities. Population studies and clinical trials suggested a survival benefit for patients taking statins during infection ^157^, which prompted interest in the ability of these drugs to modulate immune function. *In vitro* simvastatin treatment has been shown to have beneficial effects on migration of neutrophils from patients with COPD ^158^ and during pulmonary infection in otherwise healthy older adults but not during more severe infection or sepsis ^159^. These beneficial effects on migration were replicated in a clinical trial of high-dose simvastatin in healthy older adults ^159, 160^. Similar *in vivo* studies are required to determine whether effects are maintained in a disease setting, but a clinical trial is currently under way ^161^ and outputs are expected this year. Other commonly used drugs may also provide mechanistic insight into how neutrophil could be targeted. Aspirin induces resolvin-D signalling, which has been associated with improved pneumonia outcomes in murine models ^162^, and aspirin is associated with improved survival in observational studies of pneumonia ^163^. Metformin, commonly used for glycaemic control in diabetes, has also gained interest as a potential means to target neutrophil functions, potentially modifying chemotaxis and bacterial killing through 5′ adenosine monophosphate-activated protein kinase (AMPK) activation ^164^.

Targeting local airway neutrophil apoptosis has also been suggested, and induction of airway neutrophil apoptosis reduced airway inflammation in mouse models ^165, 166^ but these models do not recapitulate all features of human disease. Effective clearance of apoptotic cells, via efferocytosis, is vital to prevent secondary necrosis and release of damaging pro-inflammatory cell contents which may heighten inflammation and contribute to further tissue damage ^167^. However, in human studies, clearance of apoptotic cells has been shown to be reduced in many inflammatory airway diseases, including COPD ^83, 84^, asthma ^168, 169^, CF and bronchiectasis ^170^. As such, induction of neutrophil apoptosis *in vivo*, without improvement of clearance mechanisms, needs to be approached with caution and may have the potential to cause more harm than good.

The final challenge is effective delivery of the desired drug to its target without impacting host defence against infection owing to neutropenia or excessive impairment of neutrophil function. Inhaled therapies may permit effective targeting of airway neutrophils whilst minimising systemic side effects; indeed, an inhaled PI3K-Δ inhibitor is in clinical trial for the treatment of COPD exacerbations (ClinicalTrials.gov Identifier: NCT03345407). The key would be to ensure penetration of the inhaled compounds into the smaller airways and newer devices offer the promise of these effects.

## Conclusions

Neutrophilic inflammation is a common feature of many airway diseases and is associated with disease progression, often irrespective of the initiating cause or underlying diagnosis. This provides a potential therapeutic target, but the target is a challenging one. The crucial role of neutrophils in clearing bacteria means that merely inhibiting their responses in a blunt or indiscriminate fashion is likely to be detrimental to the host, as demonstrated by the manifestations of neutropenia. Targeting neutrophils requires a more subtle approach. Neutrophils appear to be susceptible to epigenetic changes ^171^ which are variable but impact on function and the resultant changes appear long-lived ^172^. This might provide a mechanism for the self-perpetuating inflammation present across many airway diseases. It might be that chronic inflammation leads to epigenetic reprogramming of neutrophils, which alters their phenotype or responses. The physical damage to the lung infrastructure and especially the pulmonary vasculature might compound this by inhibiting de-priming. Unfortunately, the current evidence base for understanding neutrophil function across diseases is limited (often small studies using different techniques across different patient groups) but this is certainly worthy of more study. To ascertain whether there are shared mechanisms of neutrophil dysfunction across disease and more importantly how these might be targetable will require collaborative research across current disease silos.
